# Hepatocyte Polyploidy: Driver or Gatekeeper of Chronic Liver Diseases

**DOI:** 10.3390/cancers13205151

**Published:** 2021-10-14

**Authors:** Romain Donne, Flora Sangouard, Séverine Celton-Morizur, Chantal Desdouets

**Affiliations:** 1 Department of Oncological Sciences, Icahn School of Medicine at Mount Sinai, New York, NY 10029, USA; romain.donne@inserm.fr; 2 Liver Cancer Program, Division of Liver Diseases, Department of Medicine, Icahn School of Medicine at Mount Sinai, Tisch Cancer Institute, New York, NY 10029, USA; 3 Icahn School of Medicine at Mount Sinai, The Precision Immunology Institute, New York, NY 10029, USA; 4 Graduate School of Biomedical Sciences, Icahn School of Medicine at Mount Sinai, New York, NY 10029, USA; 5 Laboratory of Proliferation, Stress and Liver Physiopathology, Centre de Recherche des Cordeliers, F-75006 Paris, France; flora.sangouard@inserm.fr; 6 Centre de Recherche des Cordeliers, INSERM, Sorbonne Université, Université de Paris, F-75006 Paris, France

**Keywords:** hepatocytes, polyploidy, cell cycle, centrosome, DNA damage response, hepatocellular carcinoma

## Abstract

**Simple Summary:**

Polyploidy, a balanced amplification of the genome is a defining feature of the liver. Up to 90% of adult hepatocytes in rodents and around 40% of those in humans are polyploid. The polyploidy of these cells depends on both the DNA content of each nucleus (nuclear ploidy) and the number of nuclei per cell (cellular ploidy). Remarkably, the liver is one of the few mammalian organs that display changes in ploidy content during normal homeostasis, regeneration and pathological conditions. Although polyploid hepatocytes were documented over a century ago, the significance of this original phenomenon in the pathophysiology of the liver remains unclear. In this review, we focused on the mechanisms regulating hepatocyte polyploidization both during liver development and under pathological conditions. We also detailed the effects of polyploidy on liver function and explored the fate and the role of the polyploid state during chronic liver diseases.

**Abstract:**

Polyploidy, also known as whole-genome amplification, is a condition in which the organism has more than two basic sets of chromosomes. Polyploidy frequently arises during tissue development and repair, and in age-associated diseases, such as cancer. Its consequences are diverse and clearly different between systems. The liver is a particularly fascinating organ in that it can adapt its ploidy to the physiological and pathological context. Polyploid hepatocytes are characterized in terms of the number of nuclei per cell (cellular ploidy; mononucleate/binucleate hepatocytes) and the number of chromosome sets in each nucleus (nuclear ploidy; diploid, tetraploid, octoploid). The advantages and disadvantages of polyploidy in mammals are not fully understood. About 30% of the hepatocytes in the human liver are polyploid. In this review, we explore the mechanisms underlying the development of polyploid cells, our current understanding of the regulation of polyploidization during development and pathophysiology and its consequences for liver function. We will also provide data shedding light on the ways in which polyploid hepatocytes cope with centrosome amplification. Finally, we discuss recent discoveries highlighting the possible roles of liver polyploidy in protecting against tumor formation, or, conversely, contributing to liver tumorigenesis.

## 1. Introduction

Eukaryotic organisms usually contain two complete haploid sets of homologous chromosomes (diploidy, 2 n). Polyploidy, or whole-genome duplication, is the condition in which an organism or cell contains additional sets of chromosomes (e.g., 4 n, 8 n). These additional sets of chromosomes may originate from a single species (autopolyploidy) or from different, generally closely related, species (allopolyploidy). In polyploid cells, the number of chromosome may be amplified within a single nucleus (mononucleate polyploid cells), defining the nuclear ploidy, or the genetic material may be distributed between two or more nuclei (e.g., binucleate polyploid cells), defining cellular ploidy. It is important to distinguish between polyploidy and aneuploidy, which is a deviation from a multiple of the chromosome number in which one or more of the chromosomes are missing or present in excess. 

Polyploidy was first identified more than a century ago and is considered to be an evolutionary adaptation to environmental changes [1,2]. Polyploidy is confined to certain taxa and is very common in plants and in certain groups of fish and amphibians [3,4]. However, many species that are currently diploid, including humans, are derived from polyploid ancestors [5,6]. These species, who experienced ancient genome duplications followed by genome reduction, are known as paleopolyploids. The polyploidization of an entire organism is rare in mammals, in which it generally results in lethality, spontaneous abortions or embryonic resorptions [7,8]. However, the theory that polyploidy was impossible in mammals was overturned by the discovery of tetraploidy in the red vizcacha rat and its cousin, the golden vizcacha rat [9]. Whole-organism polyploidy may be rare, but polyploid cells are present at relatively high frequency in many mammalian tissues [10,11,12,13]. In physiological conditions, the transition of cells from diploidy to polyploidy is part of the developmental program in some tissues [10,14]. Polyploid cells are found in the heart (cardiomyocytes, up to 4 n), bone marrow (megakaryocytes, up to 128 n), placenta (trophoblast giant cells, up to 64 n) and liver (hepatocytes, up to 16 n). In adult tissues, injury and cellular stress can induce cell losses and tissue homeostasis is dependent on the compensation of this cell death. Polyploidization constitutes an alternative cell loss compensation strategy in postmitotic tissues lacking stem cells [12,13,15]. Compensatory proliferation mechanisms have been studied in detail in Drosophila. Losick and coworkers found that the adult abdominal epidermis responds to wounding by inducing the formation of large cells, facilitating the rapid re-establishment of an epithelial barrier [16]. Polyploidization also occurs in a sizeable proportion of human tumors [10,17,18]. Most tumors have polyploid or near-polyploid karyotypes [19,20]. Polyploidy has been shown to be associated with tumor progression, in particular due to its association with the development of chromosome instability (CIN) [21,22,23].

## 2. Polyploidization Mechanisms

**Figure 1:**
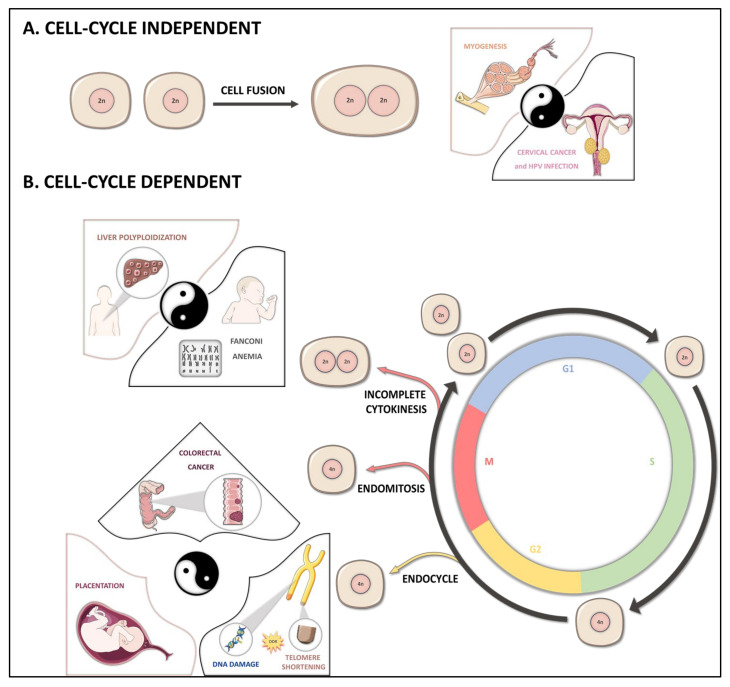
Mechanisms of physiological and pathological polyploidization in mammals. Physiological and pathological polyploid cells can be generated via different mechanisms, which may or may not be cell cycle-dependent. (**A**). Cell fusion is a cell cycle-independent process leading to the generation of binucleate/multinucleate polyploid cells. This process can occur in physiological conditions, through receptor-ligand interactions, during myoblast differentiation (pink box), for example. Infections with diverse viruses can also induce cell fusion and promote the formation of pathological polyploid cells, as observed during cervical cancer following infection with human papillomavirus (black box). (**B**). Other mechanisms are based on disturbances of the normal eukaryotic cell cycle, during interphase or mitosis progression. The first such mechanism, endoreplication, includes two types of cell cycle modifications: the endocycle and endomitosis, both of which lead to the generation of monucleate polyploid cells. During the endocycle, cells alternate between the G and S phases, and mitosis is completely shut down. This process plays a major role in the differentiation of trophoblast giant cells, a very important step in placentation (pink box, bottom). In response to telomere shortening and DNA damage, persistent activation of the DDR (DNA damage response) results in G2-M arrest and the emergence of pathological polyploid cells. Endomitosis is a variant of mitosis without nuclear (karyokinesis) and cytoplasmic (cytokinesis) division. Endomitosis has been observed to occur in cells after prolonged mitotic arrest in response to spindle toxins and in APC-deficient cells (APC = adenomatous polyposis coli, a gene frequently mutated in colon cancer; black box, middle). The second mechanism is cytokinesis failure. During this process, cells achieve karyokinesis but skip cytokinesis, leading to the formation of binucleate polyploid cells. A very good example of physiological cytokinesis failure is the progressive polyploidization of hepatocytes during liver maturation (pink box, upper). Cytokinesis failure is also associated with several diseases, such as Fanconi anemia (black box, upper).

One fascinating question concerns the mechanisms by which diploid organisms develop polyploid cells. Polyploid cells may be generated in a physiological or pathological state, by cell cycle-dependent or -independent mechanisms (Figure 1).

### 2.1. Cell Fusion

Cell fusion is cell cycle-independent and may involve cells of the same type (homotypic fusion) or cells of different origins (heterotypic fusion). It involves several steps, beginning with cell-cell adhesion, followed by destabilization of the membrane lipid bilayer, fusion and pore formation in the two cells. In some cell types, such as skeletal muscle cells and bone osteoclasts, cell fusion is a normal programmed step in development, resulting in the production of terminally differentiated polyploid cells (Figure 1A) [12,24]. Cell fusion may also occurred during pathological processes. It may be caused by viral infection, for example [25]. The well-known example of this is human papillomavirus (HPV) infection, which has been identified as an etiology of cervical cancer (Figure 1A). Indeed, infection promotes the fusion of infected cells, leading to the formation of binucleate tetraploid progenies, a common characteristic of precancerous cervical lesions [26].

### 2.2. Endoreplication

In mammals, the canonical cell cycle comprises four steps: G1 (growth), S (DNA/centrosome duplication), G2 (growth and preparation for entry into mitosis) and M (mitosis/cell division) (Figure 1B). The entry into and progression through each step are tightly regulated by a number of different proteins, including cyclin-dependent kinases (CDKs) and cyclins, the catalytic activity of which is stimulated by tight binding to CDKs. Polyploid cells can be generated by a mechanism known as endoreplication (or endoreduplication) in which DNA replication is uncoupled from cell division [11,27,28]. Endoreplication encompasses both endocycling and endomitosis (Figure 1B). In both cases, the resulting cellular progenies are mononucleate polyploid cells. Cells undergoing endocycling repeatedly alternate between the G and S phases without cell division (Figure 1B). Entry into mitosis is inhibited by two key events: (a) proteolysis of the mitotic cyclin B1 and the inhibition of mitotic-CDKs; (b) tight regulation of S-CDK activity (low levels of activity in G phase and high levels in S phase), re-inducing genome replication [12,27]. Endocycles occur in bacteria, green algae, protists and multicellular organisms, including plants and animals [27]. They are rarer in mammals, although the differentiation of trophoblast stem cells into trophoblast giant cells (64 n to 1024 n), of crucial importance for implantation and the modulation of post-implantation placentation, is a well-characterized example. A very elegant study recently showed that endocycling is also induced as an alternative to tissue repair. Following acute kidney injury, most of the tubular epithelial cells undergo endocycling and become hypertrophied, an event associated with a better recovery of kidney function after injury [29].

In recent years, much attention has been paid to defining the connection between the DNA damage response (DDR) and endoreplication. A link between ATM/ATR (ataxia-telangiectasia-mutated and ataxia-telangiectasia-Rad3-related)-dependent pathways and endoreplication cycles has been conserved during evolution [11]. For example, the root tip and sepal cells of *Arabidopsis* display an activation of ATM and ATR orthologs and endoreplication following the induction of double-stranded DNA breaks [30]. In mammals, telomere shortening also appears to be a potent endoreplication-promoting mechanism. Indeed, in p53-null mouse embryonic fibroblasts, DNA damage signals due to persistent telomere dysfunction were shown to activate DDR in the G2 phase, leading to irreversible cell cycle arrest [31,32]. The affected cell then exit replication without undergoing mitosis, resulting in the formation of tetraploid mononucleate cells. In all these cases, endoreplication is considered to be a clear strategy for sustaining growth under genotoxic stress.

During endomitosis, cells with duplicated DNA undergo an abortive mitosis (Figure 1B). This process has been particularly well described in cultured cells, mostly displaying a prolonged arrest in metaphase due to incorrect attachment of the spindle microtubules to the mitotic chromosomes. In the presence of such incorrect attachment, the spindle assembly checkpoint (SAC) is activated and prevents passage through anaphase. The cells then either die during mitosis (mitotic catastrophe) or slip out of mitotic arrest (mitotic slippage) to generate tetraploid progenies [33,34]. Slippage is thought to occur through a gradual proteolysis of cyclin B1 [35]. This process has been described in colorectal cancer cells with mutations of the *APC* (adenomatous polyposis coli) gene, which encodes a protein crucial for the maintenance of mitotic microtubule spindles [36]. Conversely, microtubule agents are widely used in first-line treatments for several cancers. Tubulin-binding agents used in this context, such as taxane, vinca alkaloids and docetaxel, can activate the SAC in cancer cells [37,38]. Following slippage, the cells follow one of two principal fates: cell death during mitosis or entry into the next interphase as tetraploid cells at risk of G1 arrest leading to senescence. The tetraploid contingent has been shown to be able to elicit the senescence-associated secretory phenotype (SASP) and to confer paracrine protumorigenic effects, such as cell proliferation, migration, invasion and angiogenesis. These effects may contribute to the emergence of acquired chemoresistance [39].

### 2.3. Cytokinesis Failure

Cytokinesis is the last step in cell division. It is tightly regulated to ensure the correct inheritance of both the nuclear and cytoplasmic content of the parent cell by the two nascent daughter cells. This process proceeds via four steps: establishment of the division plane, contraction of the actomyosin ring, ingression of the cleavage furrow and, finally, cell abscission [40]. The deregulation of any one of these steps can lead to cytokinesis failure (i.e., karyokinesis without cytokinesis), also known as incomplete cytokinesis, and the formation of binucleate polyploid cells (Figure 1B). Cytokinesis failure plays an important role in the physiological differentiation and function of various human cells and tissues (e.g., heart, bone marrow, liver) (Figure 1B) [41]. A fascinating example of cytokinesis failure is provided by the generation of binucleate secretory alveolar cells in the lactating mammary gland. These cells first appear in late pregnancy. An increase in the production and/or activity of the mitotic kinases Aurora kinase-A and Polo-like kinase-1 at the lactogenic switch probably mediates the formation of binucleate cells [42]. Binucleation is advantageous for secretory alveolar cell function, as it results in a greater surface area, leading to more efficient milk secretion [42]. Cytokinesis failure has also been implicated in various diseases, including hemopathies, female infertility syndrome, age-related macular degeneration and cancers (Figure 1B) [18,41]. In these diseases, cytokinesis failure may result from various events, such as a failure to resolve chromatin bridges, ultrafine DNA bridges or the lagging chromosome in the cleavage furrow. Moreover, mutations or changes in the expression of genes encoding proteins responsible for regulating cytokinesis initiation and progression (e.g., ECT2, RHOA, LATS1) have been shown to cause cytokinesis failure, particularly in precancerous cells [18].

## 3. The Liver: A Singular Polyploid Organ

The liver is a crucial organ in the human body, as it fulfills a myriad of functions supporting metabolism, immunity, digestion, detoxification, and vitamin storage, for example. The adult liver contains both parenchymal and non-parenchymal cells. The parenchymal cells are the hepatocytes, which account for 60% of all liver cells and 80% of the weight of this organ. The non-parenchymal cells consist of liver endothelial cells (LECs), hepatic stellate cells, biliary epithelial cells (cholangiocytes), Kupffer cells and additional immune cell populations [43]. The liver is also unique in having a dual blood supply, with blood arriving via both the portal vein and the hepatic artery, creating gradients of nutrients, oxygen, hormones and gut-derived endotoxins that collectively shape the molecular and functional heterogeneity of hepatocytes in a well-known process called liver zonation [44,45]. Some very elegant single-cell RNA sequencing studies have recently shed new light on the molecular nature of this heterogeneity [46]. Processes that are energetically demanding, such as protein secretion and gluconeogenesis, are allocated to the portal layers, where oxygen is more abundant. Mid-lobule hepatocytes specialize in several different tasks, including secretion of the iron-regulating hormone hepcidin. Pericentral hepatocytes are involved principally in xenobiotic metabolism, bile acid biosynthesis and glycolysis, which are less energetically demanding processes. Hepatocytes have a half-life of about 200 days in rodents and 400 days in humans, explaining the quiescent nature of these cells [47,48]. Polyploidy is also a characteristic feature of mammalian hepatocytes that was discovered a long time ago. The polyploidy of these cells depends on both the DNA content of each nucleus (nuclear ploidy) and the number of nuclei per cell (cellular ploidy) [24,49]. For example, polyploid hepatocytes may be tetraploid [e.g., binucleate with two diploid (2 n) nuclei or mononucleate with a single tetraploid (4 n) nucleus] or octoploid [e.g., binucleate with two tetraploid (4 n) nuclei or mononucleate with a single octoploid (8 n) nucleus]. Several studies have determined the proportions of polyploid hepatocytes in the liver parenchyma of different species. Up to 90% of adult hepatocytes in rodents [50,51] and around 40% of those in humans [52,53] are polyploid. However, hepatocyte polyploidization is not universal. The hepatocyte of guinea pigs have a low level of cellular ploidy [54] and no polyploid hepatocytes have been detected in the normal liver tissues of woodchucks [55].

## 4. Polyploidy, an Alternative Cell Cycle Program during Development

**Figure 2:**
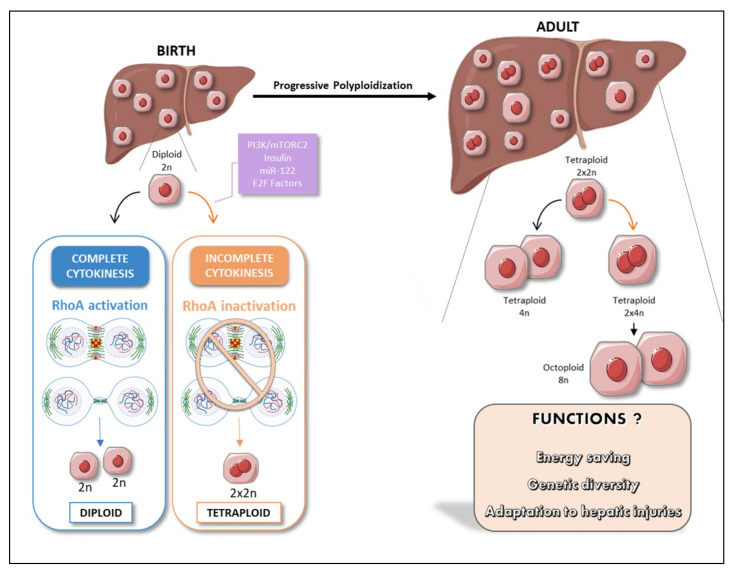
Physiological polyploidy in the liver during post-natal development. The hepatocytes of neonates are exclusively diploid (mononucleate 2 n). During weaning, diploid hepatocytes may undergo normal cell division cycle (blue box) giving rise to two diploid hepatocytes, or incomplete cytokinesis (orange box) driven by the impairment of Rho signaling, giving rise to one binucleate tetraploid hepatocyte. This developmental process is regulated by insulin, phosphoinositide 3-kinase (PI3K)-mechanistic target of rapamycin complex 2 (mTORC2), E2F and miR-122 signaling. This process leads to progressive polyploidization of the liver parenchyma during the course of postnatal development and the generation of tetraploid and octoploid hepatocytes with specific functions.

The polyploidization process has mostly been studied in rodents. At birth, all rodent hepatocytes are diploid (2 n) and have a high rate of proliferation [56]. After about three weeks of postnatal development, a subset of proliferating diploid hepatocytes perform karyokinesis correctly, but fail to complete cytokinesis, generating binucleate tetraploid daughter cells, each with two diploid nuclei (2 × 2 n) (Figure 2) [51,57]. If a binucleate tetraploid hepatocyte enters the cell cycle, it will either undergo cytokinesis, generating two mononucleate tetraploid hepatocytes (4 n), or cytokinesis will fail again, leading to the generation of a binucleate octoploid hepatocyte (2 × 4 n). This situation results in the establishment of physiological polyploidy (Figure 2). Hepatocyte polyploidization levels reach a plateau in the mature animal, and this plateau is associated with the end of liver proliferation [51]. Liver polyploidization differs between mammals, and most of the polyploid hepatocytes are binucleate cells in both rodents [58] and humans [53]. The cellular and molecular mechanisms behind this phenomenon are now well characterized. Indeed, our laboratory showed that the cytokinesis program is disrupted by an impairment of actin cytoskeleton re-organization at the division plane during the anaphase-telophase transition, resulting in the abolition of cell elongation [57]. The mitotic spindle microtubules fail to contact the cortex, and the molecular signals essential for furrow induction (e.g., AURORA B kinase and PLK1/Polo-like kinase 1) are not correctly delivered. These events impair activation of the RHO GTPase RHOA (the major orchestrator of cytokinesis) in the central cortex, leading to the generation of binucleate progenies (Figure 2) [57]. Cytokinesis failure occurs at about the time of weaning [57,58], a crucial period of development associated with profound changes in feeding behavior, metabolic pathways and hormone concentrations. We have shown that insulin is one of the key factors controlling physiological polyploidy [59,60]. Low levels of insulin in the bloodstream result in the development of fewer binucleate tetraploid hepatocytes, whereas high insulin levels promote the formation of binucleate tetraploid hepatocytes. In this context, insulin signals via the phosphoinositide 3-kinase (PI3K)-protein kinase B (Akt) mechanistic target of rapamycin complex 2 (mTORC2) cytoskeleton regulation pathway. The inhibition of PI3K/Akt phosphorylation prevents cytokinesis failure; cytokinesis is thus completed, and this event is followed by actin cytoskeleton polarization, cytoskeleton reorganization, and RhoA recruitment to the equatorial cortex [59,60]. Other factors have also been shown to play a role in postnatal liver polyploidization. E2F transcription factors—key regulators of cell cycle progression—have been implicated in the regulation of liver polyploidization [61,62,63]. The E2F family has eight known members: E2F1 to E2F8 [64]. The conditional knockout of *E2f1* leads to an increase in polyploidy, whereas the deletion of *E2f7* and/or *E2f8* prevents the generation of polyploid hepatocytes [61,62,63]. Indeed, E2F7 and E2F8 control the expression of various genes required for the execution of cytokinesis (*Ect2, Mklp1* and *Racgap1*) [61,62]. Consistent with these findings, the levels of these transcription factors increase after birth, during a period coinciding with hepatocyte polyploidization [61]. Interestingly, microRNAs (miRNAs) also drive physiological binucleation [58]. Indeed, downregulation of the expression of miR-122 (the most specific miRNA in the liver) reduces the polyploid contingent, a trend reversed by miR-122 overexpression. MiR-122 antagonizes the expression of pro-cytokinesis targets, including RhoA in particular, leading to the failure of cytokinesis and the expansion of binucleate hepatocytes.

## 5. Does Liver Polypaloidy Boost Hepatic Function?

Since the discovery of physiological polyploidy in the liver, many teams have investigated its possible consequences for the functions of the hepatic parenchyma. One of the main studies to date characterizing the specific features of polyploid contingents is based on the generation of a mouse model with a high proportion of polyploid hepatocytes in the liver. In this model, the hepatocytes lack the mitotic kinase CDK1 [65]. After partial hepatectomy, *Cdk1*-knockout livers regenerate via a specific cell cycle program, endoreplication. The authors observed that cytoskeletal gene expression levels were positively correlated with cell size, as previously demonstrated in the yeast model [66,67]. Unexpectedly, the expression levels of mitochondrial genes and genes encoding proteins involved in de novo lipid biosynthesis were found to be inversely correlated with cell size. The inhibition of mitochondrial functions and lipid synthesis increases cell size in culture, clearly suggesting causality [66]. However, it remains to be determined whether metabolic reprogramming is merely an outcome of *Cdk1* deletion. Many studies on mouse liver parenchyma have tried to determine whether polyploid cells follow a specific distribution within hepatic lobules that might account for specific functions. This issue was still unresolved, as some studies have suggested that periportal hepatocytes have a smaller polyploid contingent than perivenous hepatocytes [68,69], whereas others have suggested that the proportions of polyploid hepatocytes are similar in these two areas [57,61]. More recently, studies using three-dimensional imaging have analyzed the spatial zonation profile of murine liver ploidy. Diploid hepatocytes were found to be preferentially located and observed in the periportal region; the mid-lobular region was enriched in polyploid hepatocytes, and the perivenous region had an intermediate profile [70,71,72].

Recent developments in single-cell genomics have shed light on the possibility of populations of cells with a specific ploidy having specific functions. Katsuda et al., showed, by bulk transcriptomics and single-cell quantitative reverse-transcription polymerase chain reaction, that 2 n, 4 n and 8 n hepatocytes were globally very similar [73]. This notion was further supported by the findings of the group of Martinez-Jimenez, who nevertheless reported transcriptional differences between diploid and tetraploid cells. In this case, single-nucleus RNA-seq-2 was performed on 2 n and 4 n nuclei isolated from frozen murine livers. This very elegant study demonstrated an association of different ploidy states with different metabolic potentials, such as lipid, cholesterol and xenobiotic metabolism. It also showed that tetraploid hepatocytes adjust their gene dosage according to liver zonation, suggesting potential crosstalk between liver zonation and ploidy [74]. We now need to extend these findings to cellular ploidy (binucleate hepatocyte) and to determine whether they can be transposed to human livers.

## 6. Physiological Polyploidy in the Liver: A Strategy for Cellular Senescence?

For many years, hepatocyte polyploidy has been regarded as a sign of cellular senescence. Several groups have provided support for the hypothesis that the polyploid state suppresses growth and restricts proliferation, notably through the activation of p53 in response to acute cytokinesis failure [51,69,75,76]. Several studies have reported that polyploid hepatocyte proliferation rates are unchanged by liver injury. Thus, *E2f7/E2f8*-knockout livers, which are predominantly diploid, do not have a higher liver mass recovery rate after partial hepatectomy than wild-type livers (containing 90% polyploid hepatocytes) [61]. Similarly, polyploid hepatocytes can undergo many rounds of cell division after transplantation in a well-known model of liver repopulation, the Fah^-/-^ mouse model [77]. Wilkinson and coworkers recently showed, by both primary hepatocyte culture and liver regeneration assays, that diploid and polyploid hepatocytes responded similarly to hepatic mitogens, but that rates of entry into the cell cycle were inversely proportional to ploidy. Diploid hepatocytes entered and completed the cell cycle earlier than tetraploid hepatocytes [78]. Differences between polyploid hepatocytes were also consistently observed, with tetraploid cells entering the cell cycle more rapidly than octoploid cells [78]. The authors suggested that that there might be subtle differences in cell-cycle regulation between diploid and polyploid hepatocytes (e.g., earlier replication licensing, shorter S phase) with a major impact on proliferation potential, especially over successive rounds of cell division. In fact, the restricted proliferation of the polyploid contingent may be related exclusively to aging. Indeed, Wang and coworkers reported that senescence was rare in both octoploid tetraploid hepatocytes in young mice and in diploid hepatocytes [79]. However, the percentage of senescent, octoploid hepatocytes increased with age and the majority of aged octoploid hepatocytes expressed cell senescence markers, including p16^ink4a^ and p21 [79]. These data suggest that polyploidy may induce senescence-related changes with aging.

## 7. Liver Polyploidy and Chronic Liver Disease

The most common causes of chronic liver disease (CLD) are chronic hepatitis B or C virus (HBV/HCV) infections, alcohol abuse and non-alcoholic fatty liver disease (NAFLD) [80]. Compensatory proliferation during CLD is now known to be a major cellular process leading to polyploid adaptations and transformation events. Hepatocytes retain a unique ability to proliferate rapidly in response to aggression [81]. A continuous cycle of hepatocyte death and compensatory hepatocyte proliferation takes place during CLD. Compensatory proliferation in CLD has also been linked to polyploid adaptations because of cell-cycle defects leading to mitotic escape. For example, human liver parenchyma infected with HBV or HCV presents an increase in the polyploid hepatocyte fraction (mononucleate and binucleate) and a parallel decrease in the diploid fraction, correlated with disease severity [52,82]. During HBV infection, the HBx protein seems to play an essential role in destabilizing the hepatocyte polyploid state. In a transgenic mouse model of HBV, HBx protein production in hepatocytes has been shown to modify the timing of the cell cycle, accelerating entry into S-phase and delaying the G2-M transition [83,84]. This phenomenon triggers a transient disruption of hepatocyte polyploidization, leading to an increase in the proportion of mononucleate hepatocytes (≥4 n) at the expense of binucleate hepatocytes [84,85]. Alteration of ploidy content in HBV infected hepatocytes has been correlated with high level of DNA damage and the activation of the mitotic kinase PLK1 (Polo Like Kinase 1) [84,85]. An increase in the proportion of polyploid cells after HCV infection has been observed in human primary hepatocytes [86]. It has been shown, in hepatic cells (primary hepatocytes and HepG2 and Huh7 cells), that the core proteins of HCV can induce polyploidization by impairing the spindle assembly checkpoint function, thereby promoting mitotic segregation defects. This endomitosis process has been associated to a reduced Rb transcription and enhanced E2F-1 and Mad2 expression which results in uncoupling of mitotic checkpoint [86]. Finally, the best characterized mechanism linking compensatory proliferation and polyploidization has been described in NAFLD. Of note, NAFLD is the most common cause of chronic liver disease worldwide it has been predicted that it will become the most common underlying etiological risk factor for HCC and liver transplantation in the future [87,88,89]. We investigated the ploidy profiles during the setting of this disease [90,91]. We have observed an enrichment in tetraploid and highly polyploid mononucleate hepatocytes (≥8 n) in the fatty liver parenchyma of various NAFLD mouse models (high-fat diet, ob/ob, methionine-choline-deficient diet and PTEN^KO^) and in NASH patients. Using primary hepatocyte cultures from mouse models, we have shown that steatotic hepatocytes progress through the G1 phase, entering S phase, but that their progression through the S and G2 phases occurs later than in control hepatocytes. This delay was associated with activation of the “G2–M checkpoint” (the DNA damage response or DDR) controlled by the ATR–p53–p21 pathway, which precludes activation of the mitotic kinase CDK1–cyclin B. This mechanism leads to an endoreplication cycle and the abortion of mitosis. This work identified oxidative stress as a key driver of pathological polyploidization. NAFLD mouse models treated with an antioxidant present lower level of oxidative stress and pathological polyploidization. This work strongly suggested that the DNA damage induced by oxidative stress is the main driving factor underlying polyploidization. Interestingly, oxidative stress-induced DNA damage has been clearly identified as a prevalent signal for the induction of polyploidization in cardiomyocytes and giant granuloma macrophages [92,93]. Further studies are required to improve our understanding of the function of the hepatocyte polyploidy induced by the DDR in CLD, and to determine whether the polyploid contingent can subvert the DDR.

## 8. Polyploidy and Centrosome Amplification

The centrosome is the major microtubule-organizing center involved in polarity, migration and cell division [94]. In diploid cells, the centrosomes are a pair of centrioles embedded in a complex proteinaceous structure, the pericentriolar material (PCM). Centrosomes duplicate only once during S phase, to ensure that the cell carries two centrosomes at the onset of mitosis, enabling it to form a bipolar spindle, thereby guaranteeing balanced chromosomal segregation [95]. Polyploid cells contain extra centrosomes. More than a century ago, Boveri suggested that increases in the number of centrosomes caused cancer. It has now been clearly demonstrated that extra centrosomes cause chromosome instability, aneuploidy and trigger spontaneous tumorigenesis in multiple tissues [96,97,98]. Polyploid cells with extra centrosomes can undergo mitoses. These centrosomes may cluster together, acting as two single units mimicking a bipolar spindle, or may act as single entities, generating multipolar spindles [21,99]. Chromosome segregation errors are common during multipolar cell division and lead to random gains and losses of whole chromosome (aneuploidy). In the liver parenchyma, proliferating polyploid hepatocytes can form multipolar spindles; cell division then leads to the generation of progenies of lower ploidy, sometimes associated with aneuploidy, in a phenomenon known as “ploidy reduction” [50,100,101]. Ploidy reduction co-exists with polyploidization and re-polyploidization in injured livers [50,72]. The degree of aneuploidy in the healthy liver is still a matter of discussion [50,78,102]. However, it has been clearly demonstrated that ploidy reduction acts as an adaptation mechanism, enabling the liver to cope with chronic injury [72,78,103].

In different tissues, polyploidy is frequently associated with CIN, aneuploidy, cell transformation and tumor formation, and a number of important studies have tried to identify mechanism limiting the proliferation of polyploid cells. Pioneering studies demonstrated that tetraploidization leads to the stabilization of p53, resulting in cell cycle arrest [104,105,106]. Aneuploidization following tetraploidization is mostly observed in cells in which p53 is inactivated [76,107]. David Pellman’s group has shown that tetraploid, but not diploid, mammary epithelial cells in which p53 is inactivated give rise to malignant tumors in nude mice [107]. It is becoming clear that an aberrant number of centrosomes can trigger an antiproliferative signal in polyploid cells. Two mechanisms related to centrosome amplification and p53 activation have been reported to restrain the proliferation of the polyploid contingent. The first mechanism was deduced from the observation that large tumor suppressor kinase 2 (LATS2), the core kinase of the Hippo pathway, can be translocated from the centrosome to the nucleus, where it stabilizes p53 by inhibiting MDM2 [108]. David Pellman’s group demonstrated that cytokinesis failure, generating tetraploid hepatocytes, is a physiological activator of LATS2. LATS2 activates the p53 pathway in tetraploid hepatocytes, but it also inactivates YAP/TAZ-dependent transcription, thereby limiting proliferation [76]. Another mechanism for p53 activation in response to polyploidization was recently described. This mechanism involves a multi-protein complex, the PIDDosome. This complex was identified in 2004 as a protein complex involved in the activation of caspase-2 (CASP2) in response to genotoxic stress [109]. Since, it has been demonstrated that the PIDDosome, consisting of p53-induced death domain protein 1 (PIDD1), the adaptor protein RAIDD, and CASP2, acts as a molecular sensor of extra centrosomes, inducing p53-mediated cell cycle arrest if supernumerary centrosomes are detected [110]. PIDD1 localizes to the mother centriole. The accumulation of additional centrosomes induces assembly of the PIDDosome, through the recruitment of RAIDD and CASP2 to PIDD1. CASP2 is then activated and cleaves the E3 ubiquitin ligase MDM2, leading to p53 stabilization and p21-induced cell-cycle arrest [110]. This mechanism has been demonstrated in both cancer and non-transformed cells in which cytokinesis has failed [110]. More recently, Saldky showed, in a very elegant study, that the PIDDosome is involved in the regulation of p53 activation to limit hepatocyte polyploidy [111]. They showed that the PIDDosome-p53-p21 axis is activated in hepatocytes, in which it controls the extent of polyploidization during postnatal liver development. The PIDDosome is activated after the first round of cytokinesis failure in hepatocytes. E2F-family members regulate the production of CASP2 and PIDD1 for liver ploidy control. Interestingly, polyploid hepatocytes also engage the PIDDosome during the regeneration process, to prevent excessive polyploidization [111].

## 9. The Fate of Polyploid Hepatocytes during Liver Tumorigenesis

The occurrence of polyploid cells is a frequent signature of cancers [112,113] and whole-genome duplication in tumor tissues is strongly associated with copy number aberrations and a poor prognosis [20,114]. As previously described, several studies have shown that polyploid cancer cells emerge before the onset of aneuploidy and during the transition from premalignant to malignant lesions [26,115]. These findings strongly suggest that polyploid cells are genetically unstable and favor tumorigenesis [10,17]. The liver is physiologically polyploid, has to cope with a certain degree of aneuploidy and modifies it ploidy content following tissue injury and stress [24,101]. Deciphering the role of polyploidization in liver tumorigenesis is a major issue. Several studies have shown that human hepatocarcinomas (HCCs) are highly enriched in diploid hepatocytes, suggesting that polyploid status may protect against HCC and that diploid hepatocytes are the dominant drivers of tumorigenesis [116,117,118]. Until recently, the lack of an experimental model for manipulating ploidy levels in vivo without adverse effects for liver homeostasis hindered efforts to dissociate the tumorigenic potentials of diploid and polyploid hepatocytes. However, in recent years, great efforts have been made to develop genetic models with either low or high levels of ploidy, for careful dissection of the role of polyploidy in hepatocarcinogenesis. The groups of Leone [119] and Duncan [78] have studied the tumorigenesis process in DEN-treated *E2f7/E2f8* knockout mice. They demonstrated that these mice, in which the liver is mostly diploid, are much more susceptible to transformation than control mice, the livers of which contain a mixture of diploid and polyploid hepatocytes [78,119]. Consistent with these findings, Sladky et al., observed that PIDDosome-knockout mice with a high degree of polyploidy are protected against DEN-induced HCC, and that tumor progression in these mice seems to be driven by lower-ploidy hepatocytes [111,120]. These data provide solid evidence that the polyploid state protects the liver against tumor formation. Consistent with this conclusion, *E2f7/E2f8* transcripts [121] and some components of the PIDDosome pathway [120] have been reported to be strongly expressed in human HCC and positively correlated with a poor prognosis for patients.

**Figure 3:**
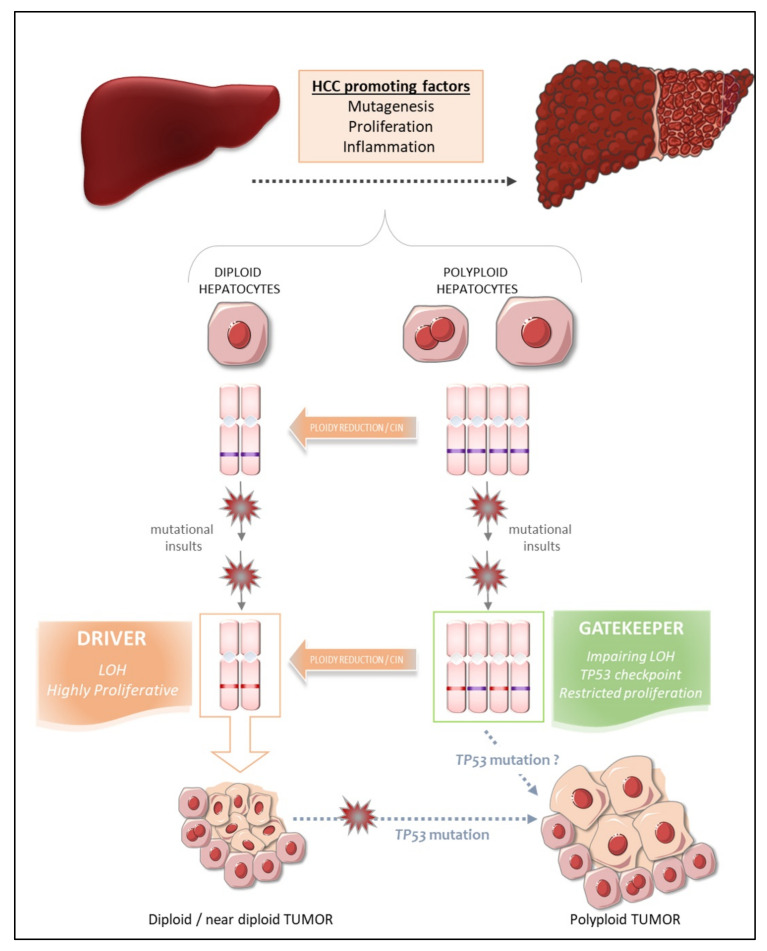
The two faces of polyploidy in HCC.Hepatocyte polyploidy can prevent tumor initiation by buffering loss of heterozygosity (LOH) and mutations of tumor suppressor genes. In addition, the limitation of proliferation mediated by the p53 checkpoint in the polyploid state, may prevent the expansion of potentially transformed clones. It is, therefore, thought that liver tumors stem from diploid hepatocytes: diploid hepatocytes exposed to multiple oncogenic insults are more prone to loss of heterozygosity. The gatekeeper phenotype of hepatocyte polyploidy can be impaired following ploidy reduction. This phenomenon can generate progenies of lower ploidy displaying chromosomal instability (CIN) that are more likely to undergo transformation following additional tumorigenic hits. *TP53* mutation may also favor the development of polyploid tumors, which are known to be more aggressive and to have a poorer prognosis.

What, then, are the intrinsic properties of polyploid hepatocytes responsible for this protection against liver tumorigenesis? Polyploid hepatocytes proliferate less efficiently than diploid hepatocytes in response to proliferative stimuli, and this may enable polyploid cells to suppress tumor development [122]. Zhu and coworkers proposed a number of other possibilities, in their work on mouse models harboring a hyperploid liver, in which they manipulated the weaning period [123] or transiently knocked down levels of the actin-binding protein anillin [123,124,125], a key factor required for cytokinesis [126]. They then explored the outcome of these hyperploid livers compared to diploid ones (loss of *E2F7/8*) in diverse cancer models. Consistent with the findings of other studies, they confirmed that mostly diploid livers were highly susceptible to HCC formation and formed abundant tumors, whereas highly polyploid livers were much less susceptible and developed few tumors [123,125]. Diploid and polyploid livers were equally susceptible to oncogene-induced carcinogenesis, but polyploid livers were more resistant to loss of heterozygosity (LOH) mechanisms mediating transformation [123]. In this context, we can speculate that the loss of one or more tumor suppressor genes by LOH in a diploid hepatocyte may favor tumorigenesis, whereas the gene redundancy inherent to polyploid genomes may prevent the initiation of HCC by providing a larger reservoir of tumor suppressor genes (Figure 3). Together, these studies support the notion that higher levels of polyploidy suppress liver tumors by limiting both loss of heterozygosity and proliferation, two of the potential drivers of transformation.

Matsumoto et al. [72] and Lin et al. [125] recently reshuffled the deck and added a level of complexity to the link between polyploidy and tumorigenesis, by revealing that polyploid hepatocytes can promote tumor initiation through ploidy reduction (Figure 3) [127]. Contrary to the studies described above, the work of these two groups directly addressed the issue of the oncogenic potential of WT diploid and polyploid hepatocytes without the genetic manipulation of ploidy status in hepatocytes. Grompe’s group developed a new multicolor reporter allele system for genetically labeling cells, to facilitate the tracing of polyploid hepatocyte fate in vivo: heterozygous Rosa-Confetti multicolor reporter mice [72,128]. In this model, diploid cells can express only one reporter, whereas polyploid cells can be labeled by the co-expression of multiple reporters of different colors, making it easy to distinguish and follow the behavior of each cell population and its progenies. They demonstrated that polyploid hepatocytes undergo a strong decrease in ploidy in response to proliferative stimuli and that polyploid hepatocytes and their ploidy-reduced daughters play a major role in the regeneration process in chronically injured livers. The regenerative nodules emerging from proliferating polyploid hepatocytes harbored chromosomal aberrations, suggesting that ploidy reduction promoted chromosome mis-segregation and chromosomal instability (CIN). Contrary to previous reports, the authors showed, in various models of hepatocarcinogenesis (oncogenic insult or tumor-prone chronic injuries), that polyploid hepatocytes per se and their ploidy-reduced daughter cells can give rise to tumors, although the frequency was very slightly lower than that of their diploid counterparts [128]. Polyploid hepatocytes undergoing continuous proliferation during serial transplantation experiments were less responsive to multipolar mitosis and, consequently, to ploidy reduction through the disappearance of their supernumerary centrosomes, than their naïve untransplanted counterparts. Serially transplanted polyploid hepatocytes had a significantly lower tumorigenic potential than these “naïve” cells. Differences in the intrinsic oncogenic properties of “naïve” and transplanted polyploid hepatocytes cannot be rule out, but these data nevertheless support the emerging hypothesis that ploidy reduction may be a step-driver in tumorigenesis [128]. Furthermore, competitive tumor formation assays have revealed that ploidy reduction during the early steps of carcinogenesis plays a greater role in models of tumorigenesis induced by tumor suppressor loss than in oncogene-induced cancer models [128]. Overall, these data highlight the possibility that proliferating polyploid hepatocytes engaged in a ploidy reduction mechanism may provide a reservoir of low-ploidy cells susceptible to both LOH and CIN, two processes widely implicated in malignant transformation [129,130]. Similarly, Lin and coworkers highlighted the potent role of ploidy reduction in liver tumorigenesis [131]. They revealed that the hyperpolyploidization of centrilobular (CL) hepatocytes after DEN exposure was associated with the emergence of premalignant lesions in the centrilobular region. Pathological polyploidization was induced by a mechanism of abscission failure dependent on upregulation of the Aurora B kinase, a master regulator of the final step of cytokinesis [132]. The prevention of DEN-induced hyperpolyploidization by the inhibition of Aurora B kinase reduced the number of preneoplastic tumor foci. Finally, as these premalignant lesions were composed of significantly smaller hepatocytes, and multipolar mitoses, which can mediate genome reduction, were observed in CL hepatocyte-derived tumor cells, the authors speculated that the phenomenon of ploidy reduction sustained the transition from hyperpolyploid hepatocytes to tumor-forming cells.

Experimental studies in mouse models can provide considerable insight into the processes linking polyploidy to tumorigenesis, as data can be obtained at both the precancerous and cancerous stages. By contrast, the opportunities for dynamic investigations of the role of ploidy in human liver HCC are quite limited. Nevertheless, some groups have carefully analyzed ploidy status in tumor tissues and the surrounding non-tumoral tissue in cohorts of patients with HCC of various etiologies, to determine whether ploidy status is a potent prognostic marker of human HCC [53,120]. We have used an in-situ imaging approach to evaluate ploidy status directly in human liver parenchyma [53]. We first showed that, in normal liver, hepatocyte polyploidy is mostly cellular (binucleate cells). No specific zonation of the polyploid contingent was observed across the lobules. We found that the binucleate polyploid fraction decreased considerably during human liver tumorigenesis, whereas the mononucleate polyploid fraction greatly increased. These results provided the first evidence to suggest that hepatocarcinogenesis can drive a conversion from physiological cellular polyploidy to pathological nuclear polyploidy. A decrease in the proportion of binucleate hepatocytes was observed in both the tumoral and surrounding non-tumoral tissues, suggesting that decreases in cellular ploidy may be a good marker of human premalignant liver parenchyma. We then carefully analyzed the nuclear ploidy profile, considering specific histological and molecular features of HCC tumors. We observed a huge increase in the percentage of 4 n and ≥8 n mononucleate hepatocytes in poorly differentiated HCC. Furthermore, taking into account the most frequent mutations in HCC, we showed that HCCs with *TP53* mutations were enriched in highly polyploid hepatocytes relative to HCCs with mutations of the TERT promoter or *CTNNB1* HCC. Finally, we found that the most aggressive HCCs, particularly those with *TP53* mutations, were enriched in highly polyploid hepatocytes. Together, these results support the hypothesis that polyploidization drives HCC development, at least in the context of *TP53* mutation, and that the nuclear ploidy spectrum can be used as a marker of HCC aggressiveness. By contrast, Sladky et al. published results suggesting that polyploidy attenuates hepatocarcinogenesis in humans. They investigated ploidy status in HCC patients by assuming that cell density (i.e., the number of cells per field) would be a good indicator of ploidy: lower-ploidy hepatocytes are small and their density is high in the liver, whereas polyploid hepatocytes are large and found at lower density [120,133]. They found that cell density in tumoral tissue tended to be lower than that in the surrounding liver and that low ploidy levels in HCC tumors (high cell density) were associated with better recurrence-free survival [120]. The conclusions of these two studies diverge, but the discrepancies may be explained by the functional status of TP53. Indeed, in a context of *TP53* mutation, polyploid hepatocytes may be able to undergo unrestricted proliferation and multipolar mitoses due to the presence of supernumerary centrosomes, facilitating the emergence of genomic aberrations/CIN, rendering the tumor more aggressive (Figure 3) [53,128]. Conversely, the presence of a functional TP53 would tend to attenuate this effect [120]. Hepatocarcinogenesis is a multistep process dependent on interplay between various factors, including risk factors, genetic factors and microenvironment factors, driving the formation of HCCs with considerable heterogeneity [134]. The assignment of a reliable and unambiguous prognostic value to ploidy status in human HCC will therefore require additional studies carefully integrating the genetic, molecular, etiological and histological landscapes of each individual tumor, and the heterogeneity within and between tumors.

## 10. Conclusions and Perspectives

As one of the few mammalian organs that are polyploid, the liver keep the unique property to modify its ploidy content following tissue injuries and stress. Studies have now revealed some role of liver ploidy during physiological and pathological context. However, we still have much to learn about this fascinating hepatocyte feature. There is a lot of questions that are still pending. Why is one ploidy state (nuclear, cellular) used over another? Do binucleate and mononucleate polyploid hepatocytes have different functions? Is there a limit to hepatocyte ploidy? Does hepatocyte ploidy influence disease progression in different pathologies? Finally, taking into account the major advances that have been performed in understanding the link between polyploidy and tumorigenesis, it will be important to understand molecular mechanisms that link these two events with the ultimate goal of patients diagnostics and/or therapies.
